# Supplementary material for: Self-assembled polyelectrolytes with ion-separation accelerating channels for highly stable Zn-ion batteries
Source: Nat Commun. 2025 Mar 8;16:2316. doi: 10.1038/s41467-025-57666-0 (PMC11890744; doi:10.1038/s41467-025-57666-0)
Supplement: Supplementary file 1 — Supplementary Information [file 41467_2025_57666_MOESM1_ESM.pdf]

Supporting information

Experimental section

Preparation of LBL self-assembly of PAH/PAA multilayers coated Zn anode

500 mg PAH (Sigma-Aldrich, average M_w ~50,000) was mixed in 10 mL distilled water to form PAH aqueous solution. PAA aqueous solution was prepared by 450 mg PAA (Sigma-Aldrich, average M_w ~450,000) and 10mL distilled water. Zn@PAH/PAA was coated by a 50 µm doctor blade, where the PAH layer was coated on the Zn foil first and dried under UV light, after rinsing with water for 5-10 min, the PAA layer was then coated on the Zn foil. Repeat above steps to obtain the PAH/PAA multilayers coated Zn foil. According to GCD curves (Figure S1), three double layers coated Zn foil exhibits the best cycling performance, so three double PAH/PAA multilayer was used to prepare the Zn@PAH/PAA in this work.

Battery Assembly

For full cell assembly, commercial MnO_2 (Sigma-Aldrich) was mixed and grounded with carbon black and PVDF at a 7:2:1 weight ratio in NMP solution. The mixture was evenly coated on a hydrophilic carbon paper with the areal loading mass around 1.5-2 mg cm^{-2}, and dried in a vacuum oven at 65 °C overnight. Afterwards, the dried cathode was assembled with the PAH/PAA multilayers coated Zn anode in a CR2032-coin cell using 2 M $ZnSO_4$ and 0.2 M $MnSO_4$ as the electrolyte. For symmetrical cell assembly, the PAH/PAA multilayers coated Zn foil was assembled with 2 M $ZnSO_4$ as the electrolyte.

Materials Characterisation

X-ray diffraction (XRD) patterns were obtained by a Bruker Vantec500 under the radiation source of Cu metal. Scanning electron microscopy (SEM) images were collected by a JEOL JSM-6701F Field Emission Scanning Electron Microscope (JEOL, Japan) at the acceleration voltage of 15 kV. The Energy-dispersive X-ray spectroscopy (EDX) images were collected by a Carl Zeiss EVO MA10 (Carl Zeiss AG, Germany) and UItim Extreme Silicon Drift Detectors (Oxford Instrumental plc, UK). Transmission electron microscope (TEM) images were carried out by a JEOL JEM-2100 Electron Microscope. Fourier transform infrared spectroscopy (FTIR) was measured by a Shimadzu IRTracer-100 with the wavenumber from 400 to 4000 cm^{-1}. The Raman data was obtained by a Thermo Scientific™ DXR3 Raman Microscope with a laser wavelength of 532 nm. The optical images and depth profile were collected by a Keyence VXH-7000N Digital Microscope, the HER reaction was observed on anode and Zn deposition was observed on cathode in the discharge process.

Electrochemical Characterisation

The long-term galvanostatic charge-discharge (GCD) test was operated by NEWARE battery testing systems. The cyclic voltammetry (CV) was measured by a VMP3 Biologic potentiostat. The electrochemical impedance spectroscopy (EIS) was tested by a Gamry Interface 1000E.

Computational Details

Density functional theory (DFT) calculations were performed using the Vienna ab initio Simulation Program (VASP). The generalized gradient approximation (GGA) method in Perdew-Burke-Ernzerhof (PBE) functional was applied to describe the exchange-correlation interaction. Conjugate gradient algorithm was employed for geometrical

optimization. The convergence criterion for the total energy and ionic force were 10^{-4} eV and 0.03 eV/Å, respectively. The cut-off energy for the plane-wave basis set was 500 eV. Monkhorst-Pack scheme was used to sample the Brillouin zone with k-point of 1×1×1 for geometrical optimization. The van der Waals (vdW) interaction was considered through DFT-D3 correction. To avoid the interlayer interactions, the vacuum layer was larger than 20 Å. To obtain the diffusion energy barriers of Zn ions on electrode surface, the climbing image nudged elastic band (CI-NEB) method was adopted. A 6×6 supercell of Zn (002) surface containing 2 atomic layers was constructed as the Zn electrode model.

The adsorption energy (E_{ads}) is defined as

$$E_{ads} = E_{total} - E_{surface} - E_{adsorbent} \quad (1)$$

Where E_{total}, $E_{surface}$, and $E_{adsorbent}$ represent the total energies of the adsorption system, the substrate, and adsorbent, respectively. It means stronger adsorption with more negative adsorption energy.

The binding energy is calculated as

$$E_b = E_{total} - \sum_i n_i \mu_i \quad (2)$$

Where n_i is the number of atoms, and μ_i is the corresponding chemical potential.

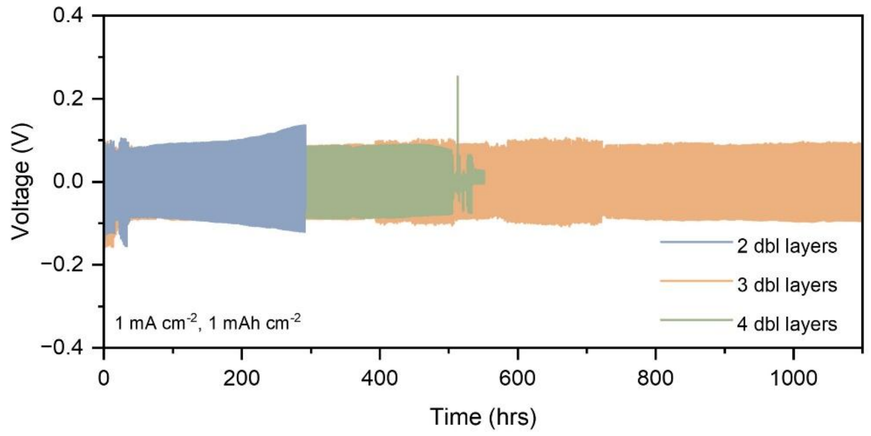

Figure S1. Galvanostatic charge-discharge (GCD) curves of the symmetric battery with different PAH/PAA layers at 1 mA cm^{-2} and 1 mAh cm^{-2}.

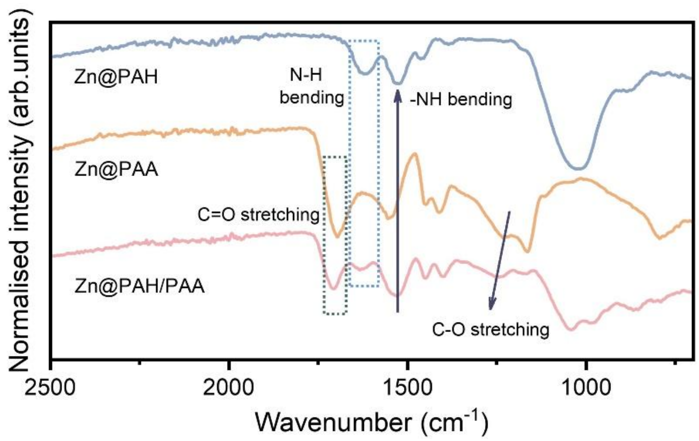

Wavenumber (cm^{-1}) / Chemical composition	Zn@PAH	Zn@PAA	Zn@PAH/PAA
C=O stretching (carboxylic acid)	N/A	1695	1710
C-O stretching (carboxylic acid)	N/A	1232	1247
N-H bending (amine)	1624	N/A	1633
-NH bending (amide)	1527	N/A	1532

Figure S2. Fourier transform infrared (FTIR) spectra of Zn@PAH, Zn@PAA, and Zn@PAH/PAA.

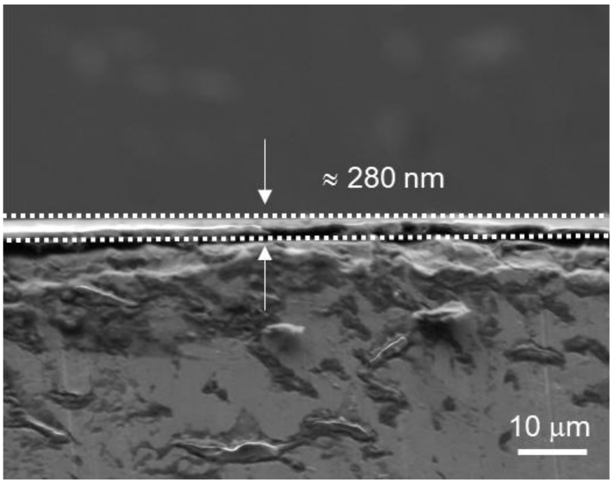

Figure S3. The cross-section SEM image of Zn@PAH/PAA before cycling.

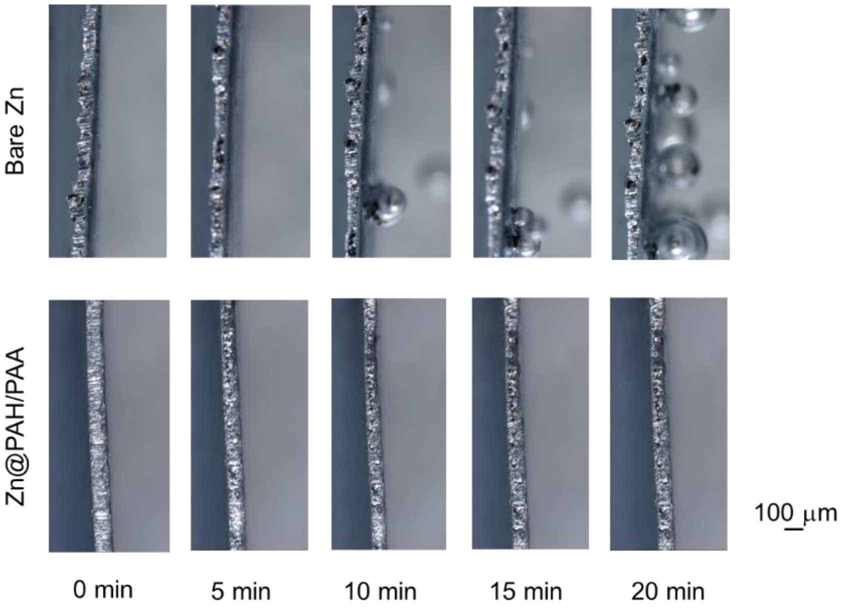

Figure S4. *In-situ* optical image of Zn stripping on Zn@PAH/PAA and Bare Zn at a current density of 30 mA cm^{-2}.

Zn@PAH/PAA electrode

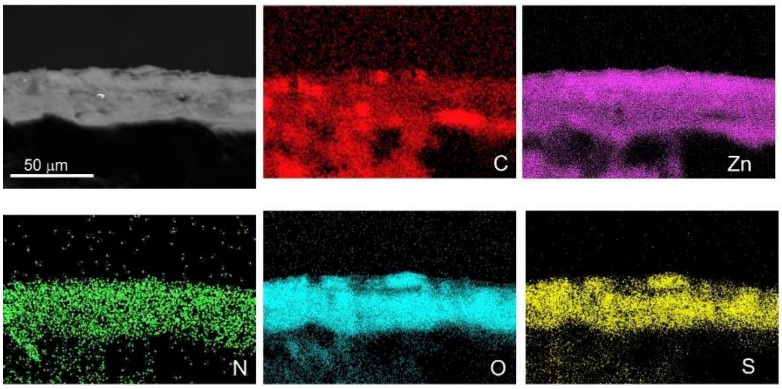

Element	At(%)
C	52.6
O	27.9
S	1.0
N	0.3
Cl	0.2
Zn	18.0
Au	0.1

Bare Zn electrode

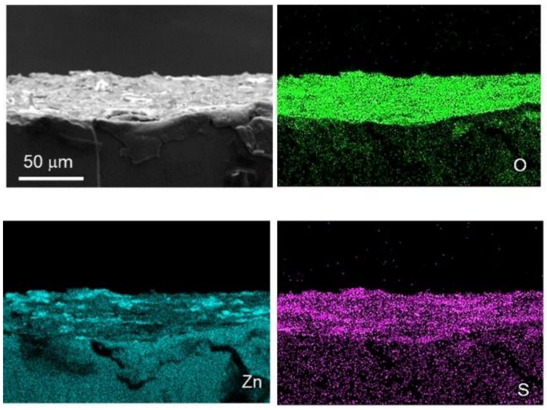

Element	At(%)
C	54.6
O	27.0
S	0.4
Zn	17.8
Au	0.4

Figure S5. EDX images of Zn@PAH/PAA and Bare Zn after 50 cycles at 0.5 mA cm^{-2} and 0.5 mAh cm^{-2}.

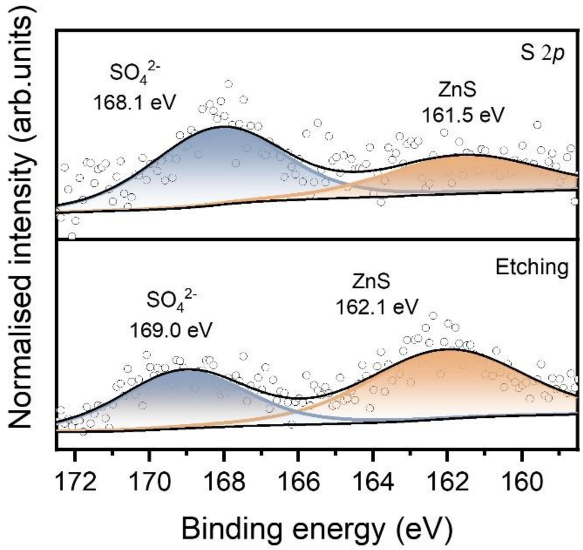

Figure S6. XPS spectra of S 2*p* for the cycled Zn@PAH/PAA electrode before and after Ar^+ etching (etching depth was set to 50 nm (TiO_2 reference)). (Cycling parameter: 50 cycles at 0.5 mA cm^{-2} and 0.5 mAh cm^{-2}).

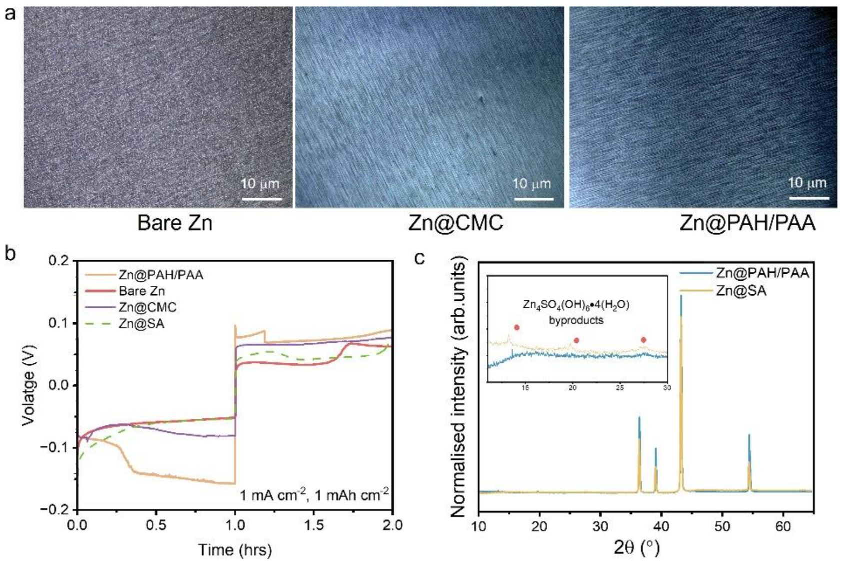

Figure S7. (a) Optical image after coating in mono and multilayers. (b) Zn||Zn performance at 1 mA cm^{-2} and 1 mAh cm^{-2}. (c) XRD comparisons after 50 cycles at 1 mA cm^{-2} and 1 mAh cm^{-2}.

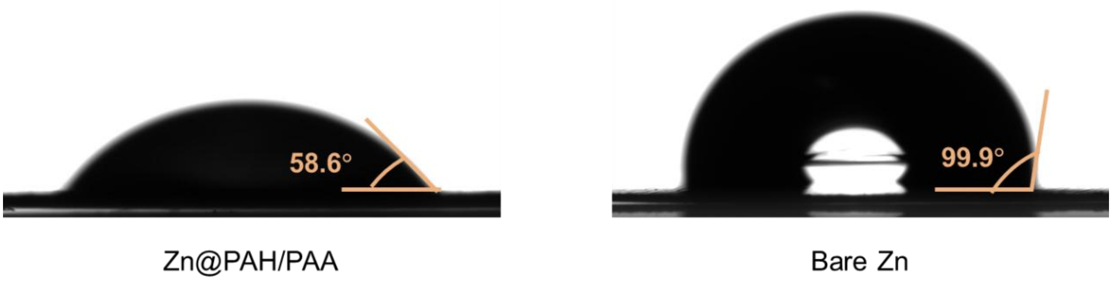

Figure S8. Contact angle test of 2M $ZnSO_4$ on Zn@PAH/PAA and Bare Zn.

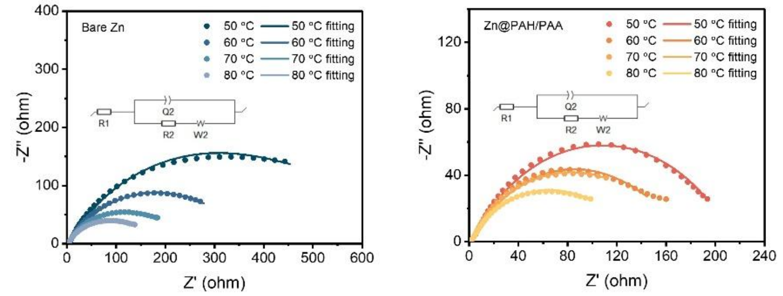

Zn@PAH/PAA	R1 (ohm)	R2 (ohm)	Fitting error
50 ºC	1.548	212.1	0.1553
60 ºC	1.345	164.3	0.1567
70 ºC	1.263	153.5	0.1246
80 ºC	1.205	124.7	0.056

Bare Zn	R1 (ohm)	R2 (ohm)	Fitting error
50 ºC	2.598	553.8	0.4275
60 ºC	2.205	342.1	0.3236
70 ºC	1.942	213.8	0.1795
80 ºC	1.74	159.8	0.093

Temperature (℃)	50	60	70	80
Bare Zn R_{ct} (ohm)	553.8	342.1	213.8	159.8
Zn@PAH/PAA Rct (ohm)	212.1	164.3	153.5	124.7

R1: internal resistance R2: Charge transfer resistance (Rct)

Figure S9. EIS test at different temperatures of Zn@PAH/PAA and Bare Zn.

Zn transference number was calculated by the equation as below, where the potentiation polarisation (∇V) is 5 mV, R_o and R_{ss} are the initial and steady state resistances, and I_0 and I_{ss} are initial and steady currents, respectively.

$$t_+ = \frac{(\nabla V/I_0 - R_0)}{(\nabla V/I_{ss} - R_{ss})}$$

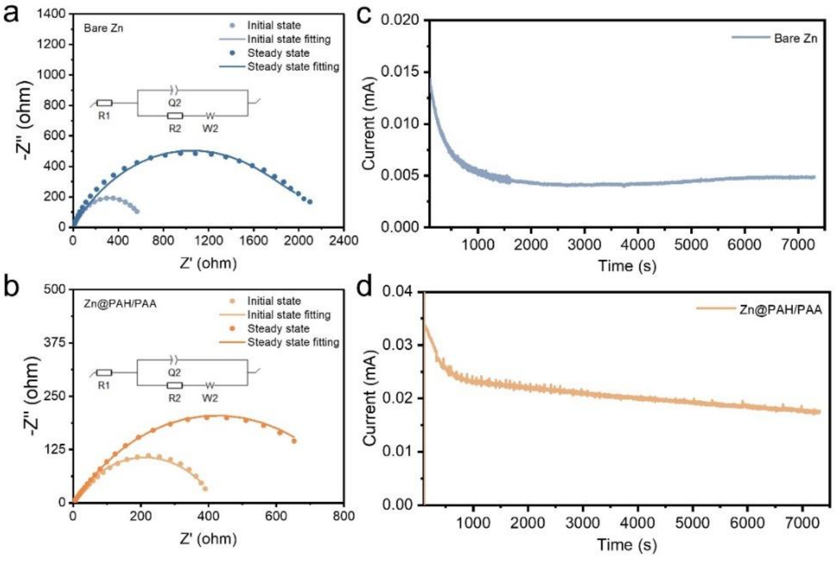

Bare Zn	R1 (ohm)	R2(ohm)	Fitting error
Initial state	0.0598	637.7	0.7296
Steady state	0.04	2068.94	1.397

Zn@PAH/PAA	R1(ohm)	R2(ohm)	Fitting error
Initial state	1.506	418.3	0.2295
Steady state	2.695	841.2	0.1082

R1: internal resistance R2: Charge transfer resistance (Rct)

Figure S10. Zn transference number calculation. EIS test at before and after the potentiostatic polarisation for Bare Zn (a) and Zn@PAH/PAA (b). Current profile under the potentiostatic polarisation (∇V= 5 mV) for Bare Zn (c) and Zn@PAH/PAA (d).

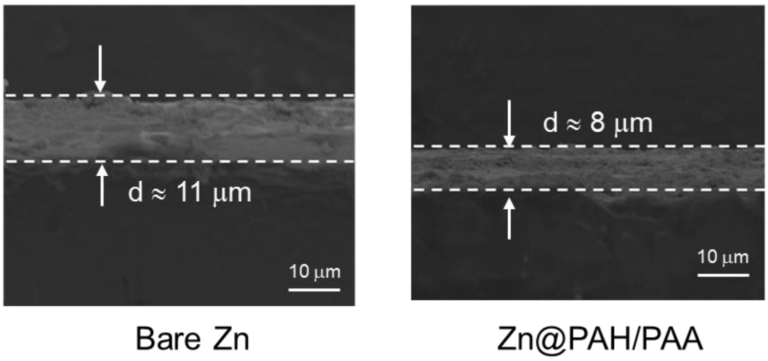

Figure S11. Cross-section SEM images after 50 cycles at 0.5 mA cm^{-2} and 0.5mAh cm^{-2}.

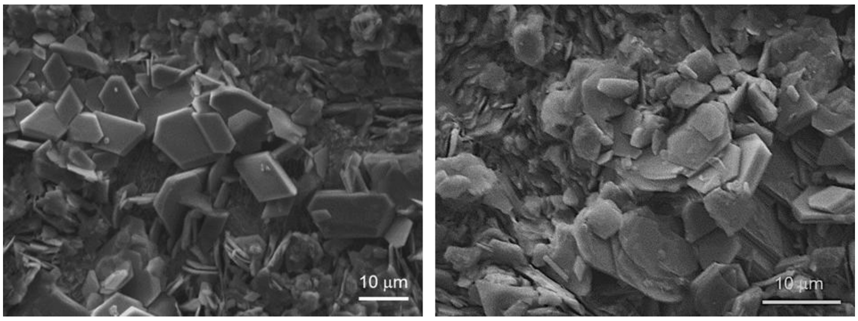

Figure S12. SEM images of Bare Zn after 50 cycles at 0.5 mA cm^{-2} and 0.5mAh cm^{-2}.

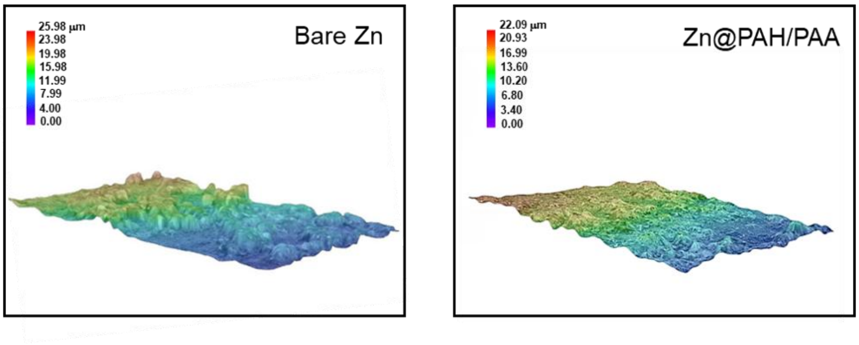

Figure S13. 3D depth profiles of Zn@PAH/PAA and Bare Zn after 50 cycles at 0.5 mA cm^{-2} and 0.5mAh cm^{-2}.

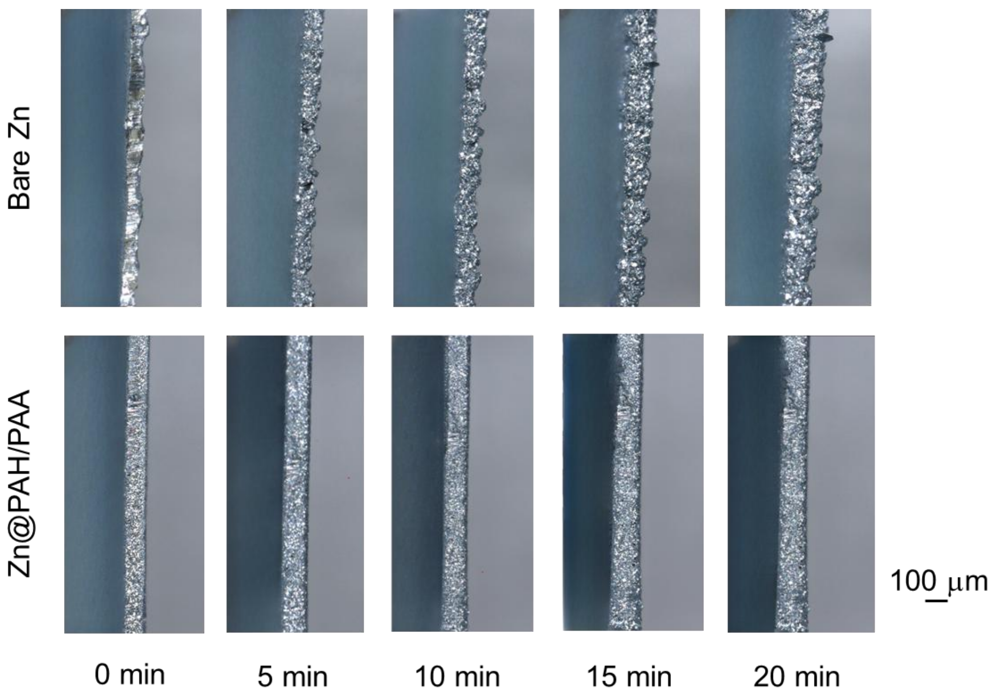

Figure S14. *In-situ* optical image of Zn plating on Zn@PAH/PAA and Bare Zn at a current density of 30 mA cm^{-2}.

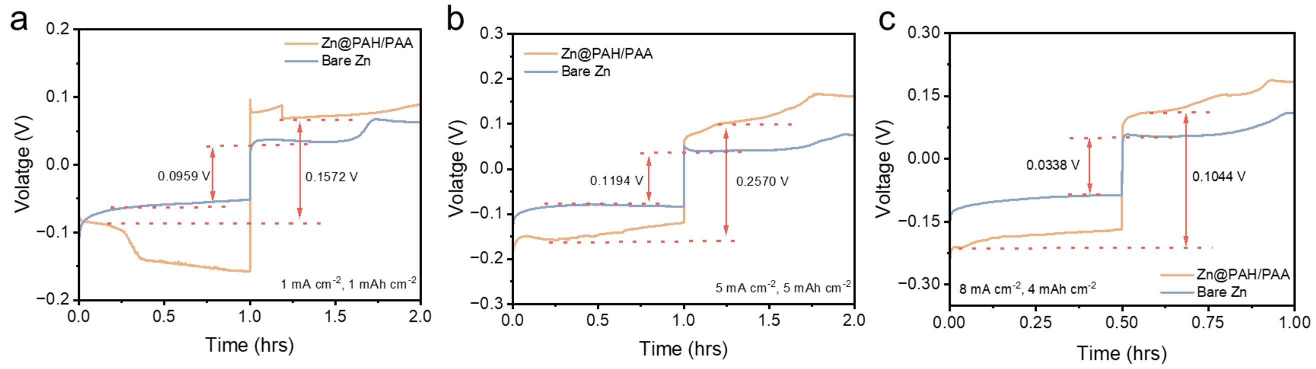

Figure S15. The voltage profile of the symmetric Zn cell at 1 mA cm^{-2} and 1 mAh cm^{-2} (a), 5 mA cm^{-2} and 5 mAh cm^{-2} (b), and 8 mA cm^{-2} and 4 mAh cm^{-2} (c).

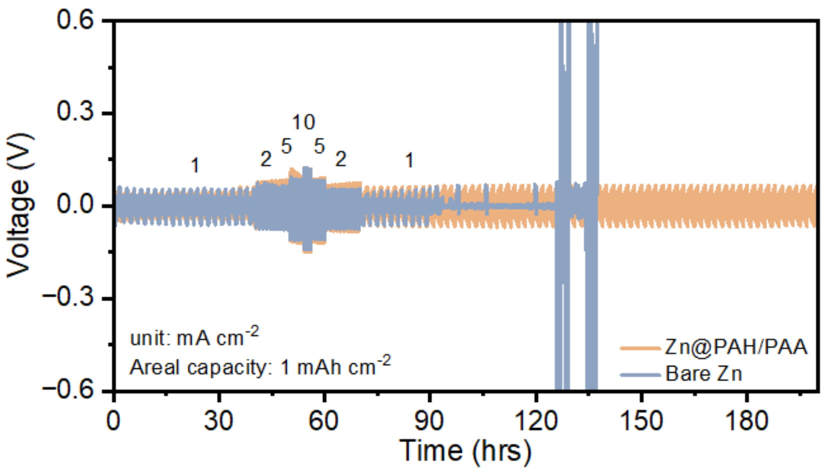

Figure S16. The rate performance of the symmetric Zn cell.

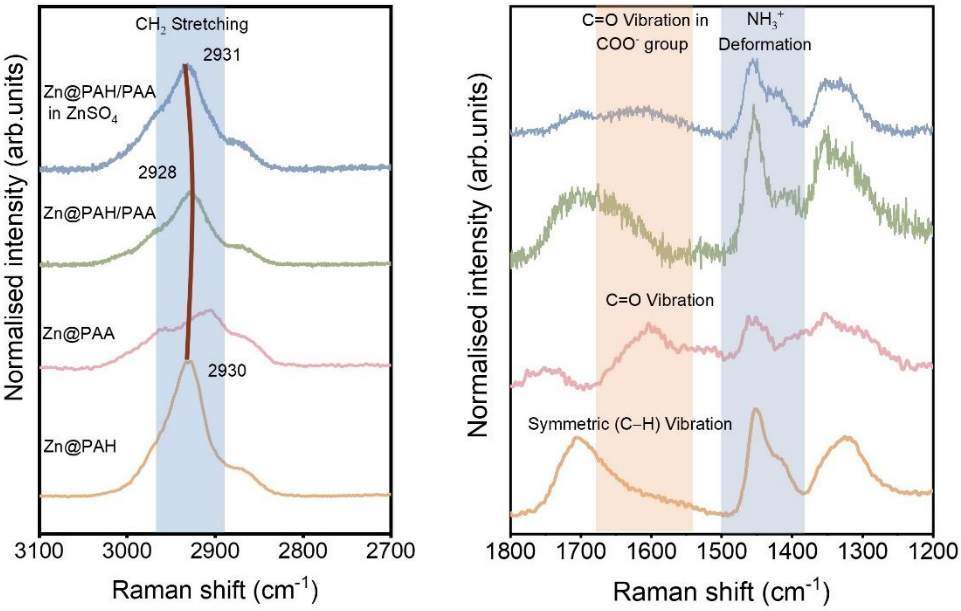

Figure S17. *In-situ* Raman spectra of different substrates.

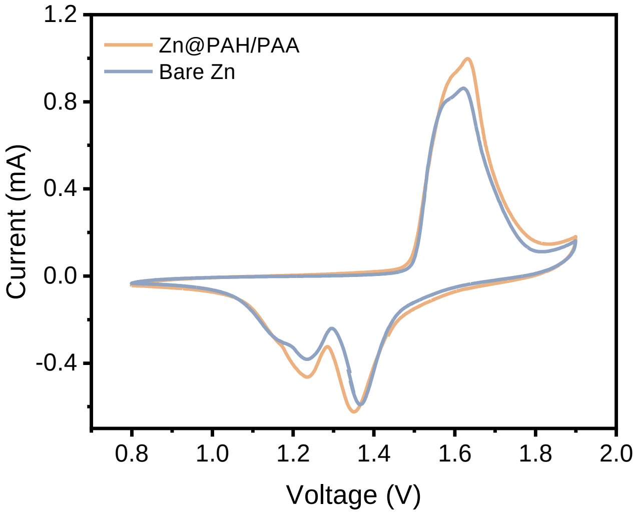

Figure S18. CV curves at a scan rate of 0.1 mV s^{-1} for the Zn-MnO_2 full cell.

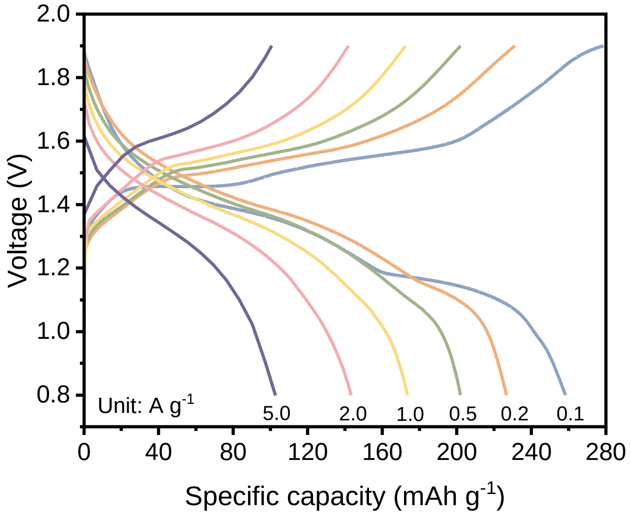

Figure S19. The correspond voltage-capacity profile at different current density.

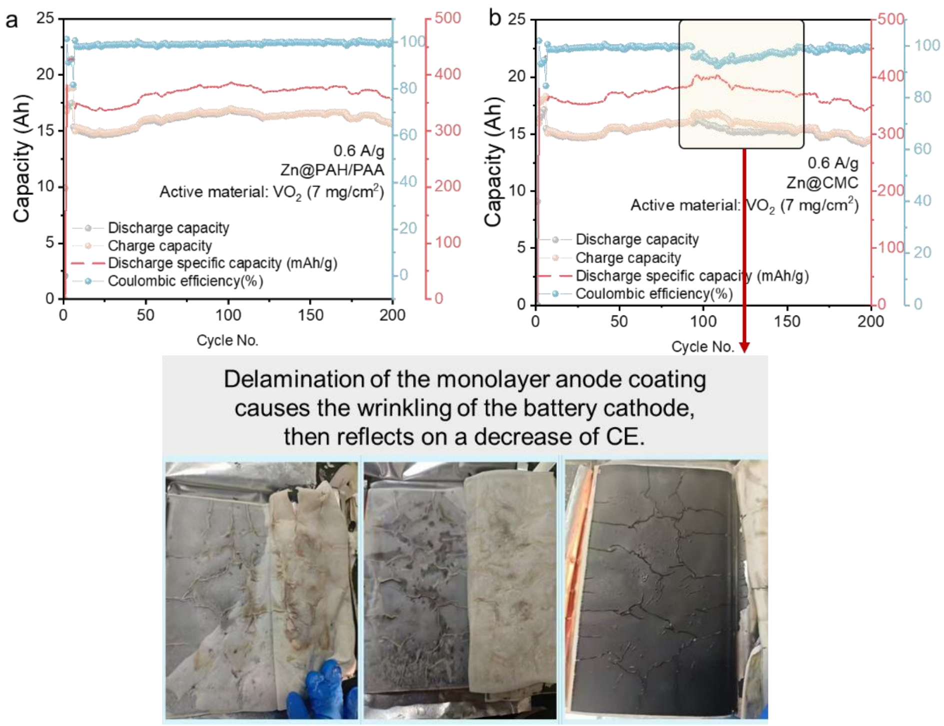

Figure S20. (a) Pouch cell of Zn@PAH/PAA in 15Ah; (b) Pouch cell of Zn@CMC in 15Ah (Anode coating layer delamination).

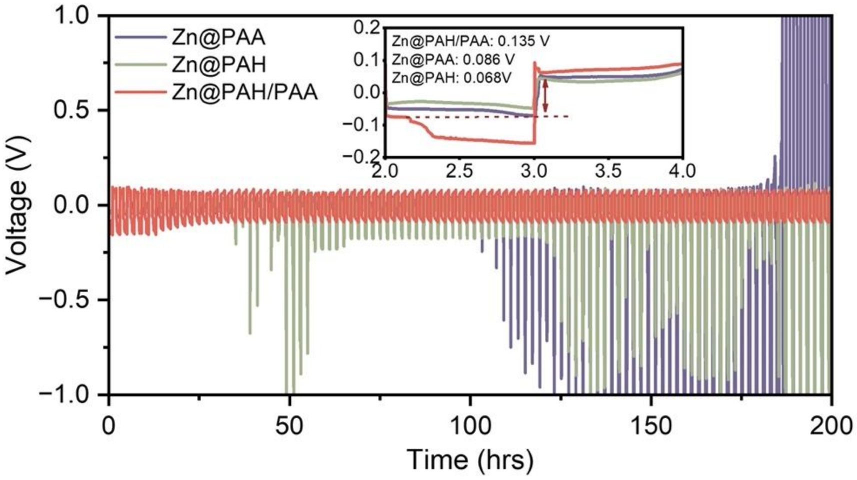

Figure S21 The galvanostatic cycling performances of Zn symmetric cells at 1 mA cm^{-2} and 1 mAh cm^{-2} with coatings of PAH/PAA multilayers, PAA monolayer, and PAH monolayer, respectively.

Table S1 XRD Intensity.

Plane	Pristine	15 cycles	30 cycles	50 cycles
002	46.94	69.96	126.92	190.96
100	81.66	107.3	78.44	106.26
101	768	638.78	552.14	642.14

Table S2 Comparison of recent anode performance on CPC, cycle number and average CE.

Reference	Sample (coating layer)	Test condition	Cycle number	Cumulative plated capacity (mAh cm^{-2})	Average CE (%)
43	CCF (cerium based conversion film)	1.13 mA cm^{-2};0.57 mAh cm^{-2}	120	68.4	99.1
44	ZCS ($Zn3(PO_4)_2$ and ZnF_2 composite SEI layer)	4 mA cm^{-2};2 mAh cm^{-2}	90	180	99.54
45	PVA@SR-$ZnMoO_4$	0.5 mA cm^{-2};0.5 mAh cm^{-2}	~500	~250	99.42
46	ZnS	2 mA cm^{-2};1 mAh cm^{-2}	200	200	99.2
47	ZnO(3D)	2 mA cm^{-2};0.5 mAh cm^{-2}	300	150	99.55
48	PVB(poly(vinyl butyral))	4 mA cm^{-2};2 mAh cm^{-2}	110	220	99.4
This work	PAH/PAA	0.5 mA cm^{-2};0.25 mAh cm^{-2}	1585	396	99.7

Table S3 The periodic band changes at $-CH_2$ and C=O groups.

CH_2 stretching						
Cycle	1st Dis	1 st Chr	2nd Dis	2nd Chr	3rd Dis	3rd Chr
Raman shift (cm^{-1})	2925.8	2929.1	2926.4	2929.9	2924.4	2928.2

C=O vibration						
Cycle	1st Dis	1 st Chr	2nd Dis	2nd Chr	3rd Dis	3rd Chr
Raman shift (cm^{-1})	1609.9	1616.7	1594.5	1604.7	1599.6	1604.2

Table S4 The binding energy of different coordination structure of PAH^+.

Coordination	Binding energy (eV)
$ZnSO_4$	-3.785
$[Zn(SO_4)_2]^{2-}$	-10.069
$[(PAH)_3SO_4]^+$	-18.766
$[(PAH)_3Zn(SO_4)_2]^+$	-26.33
$[(PAA)_1SO_4]^{3+}$	-7.384

Table S5 Comparison of anode performance regarding Zn metal pouch cells on capacity, cycle number and C-rate.

Reference	Material	Capacity (Ah)	Specific current (A g^{-1})	Cycle number
55	VO_2	0.8	0.1	200
56	$Zn_{0.25}V_2O_5{\cdot}nH_2O$	2.3	0.044	160
57	$Zn_xV_2O_5{\cdot}nH_2O$	0.9	0.2	200
58	Commercial VOX	2.7	0.05	220
59	$V_2O_5{\cdot}nH_2O$	1.32	0.1	65
This work	VO_2	17.36	0.43	250

Table S6 Cost breakdown for the LbL self-assembled PAH/PAA multilayers strategy.

Item	Unit Price	Quantity Required	Total Cost	Notes
Poly(allylamine hydrochloride) (PAH)	\$6.1 / g	2 g per m² coating	\$12.20 / m²	Based on ¥218.0 / 5 g; molecular weight ~50,000.
Poly(acrylic acid) (PAA)	\$0.98 / kg	5 g per m² coating	\$0.001 / m²	Economical, bulk industrial price of PAA at ¥7,000 per ton.
Deionized Water (Solvent)	\$0.007 / L	2 L per m² coating	\$0.014 / m²	Environmentally friendly solvent used for LbL assembly.
Processing (Roll-to-Roll Coating)	\$0.56 / m²	1 m² coating area	\$0.56 / m²	Estimated using industrial roll-to-roll coating costs.
Energy Costs (Drying)	\$0.11 / m²	1 m² coating area	\$0.11 / m²	Includes heating and drying during the LbL assembly process.
Total (Lab scale)	\$12.87/ m²			
Total (Industrial Scale)	\$4-6.40/m²			50-70% price reduction
