# Supplementary material for: Self-assembled polyelectrolytes with ion-separation accelerating channels for highly stable Zn-ion batteries
Source: Nat Commun. 2025 Mar 8;16:2316. doi: 10.1038/s41467-025-57666-0 (PMC11890744; doi:10.1038/s41467-025-57666-0)
Supplement: Supplementary file 2 — Description of Additional Supplementary Files [file 41467_2025_57666_MOESM2_ESM.pdf]

Description of Additional Supplementary Files

File name: Supplementary Dataset

Description:

Supplementary Data 1 Structural file for initial state of Zn2+ diffusion on Zn (002) crystal plane without PAH/PAA multilayers
Supplementary Data 2 Structural file for finial state of Zn2+ diffusion on Zn (002) crystal plane without PAH/PAA multilayers.
Supplementary Data 3 Structural file for initial state of Zn2+ diffusion on Zn (002) crystal plane with PAH/PAA multilayers.
Supplementary Data 4 Structural file for finial state of Zn2+ diffusion on Zn (002) crystal plane with PAH/PAA multilayers.
