# Supplementary material for: Self-assembled polyelectrolytes with ion-separation accelerating channels for highly stable Zn-ion batteries
Source: Nat Commun. 2025 Mar 8;16:2316. doi: 10.1038/s41467-025-57666-0 (PMC11890744; doi:10.1038/s41467-025-57666-0)
Supplement: Supplementary file 3 — Supplementary Dataset [file 41467_2025_57666_MOESM3_ESM.pdf]

Supplementary Data 1 Structural file for initial state of Zn^{2+} diffusion on Zn (002) crystal plane without PAH/PAA multilayers.

```
POSCAR
1.0
       15.9893999100         0.0000000000         0.0000000000
       -7.9946999550        13.8472265133         0.0000000000
        0.0000000000         0.0000000000        25.0000000000
   Zn
   73
Direct
     0.055220060         0.110843353         0.427148819
     0.109330416         0.053654719         0.527472854
     0.221226484         0.109963074         0.428075671
     0.276917577         0.053665362         0.527564049
     0.388092667         0.109508544         0.428097069
     0.443425119         0.053676270         0.527574718
     0.555404127         0.109966621         0.428085595
     0.610991299         0.053674545         0.527480662
     0.722293317         0.110842571         0.427156746
     0.778166533         0.055748194         0.527108550
     0.888886869         0.111110948         0.426667482
     0.944235086         0.055748805         0.527107835
     0.055218287         0.277710646         0.427149206
     0.108569026         0.220949486         0.527539074
     0.220950902         0.277142972         0.428740263
     0.271264553         0.216235369         0.528479338
     0.387738496         0.275677025         0.428685158
     0.443391353         0.220127627         0.529134929
     0.554619908         0.275695652         0.428711623
     0.611629665         0.216274381         0.528484941
     0.722856224         0.277143478         0.428755224
     0.779030800         0.220954835         0.527543008
     0.889155030         0.277708113         0.427154034
     0.944233477         0.221813813         0.527105272
     0.055586427         0.444513053         0.426381201
     0.109329827         0.389003634         0.527472556
     0.221224710         0.444599181         0.428075373
     0.271265358         0.388357520         0.528481066
     0.387735337         0.445393980         0.428685635
     0.428240031         0.380783111         0.521996021
     0.555552304         0.444442004         0.433550447
     0.619186044         0.380857795         0.522066116
     0.724307895         0.445380777         0.428702742
     0.783733726         0.388369888         0.528491199
```

0.890030980	0.444597125	0.428080648
0.946316779	0.389004797	0.527475536
0.055819046	0.611146450	0.426927269
0.111518972	0.556374848	0.527312219
0.221987650	0.611148655	0.426925123
0.276916742	0.556579590	0.527565718
0.388089210	0.611915410	0.428097844
0.443391353	0.556591094	0.529138327
0.554615021	0.612254322	0.428711116
0.619190395	0.571658373	0.522064149
0.724308074	0.612255931	0.428702533
0.779885471	0.556605518	0.529145837
0.890480340	0.611904144	0.428100854
0.946324825	0.556574643	0.527572513
0.055819124	0.778007567	0.426928163
0.111502707	0.722413778	0.526552439
0.222219944	0.777777255	0.427424133
0.277582586	0.722416282	0.526555657
0.388855040	0.778011739	0.426927984
0.443422526	0.723073244	0.527573764
0.555402279	0.778767407	0.428086936
0.611628652	0.728683531	0.528486252
0.722854495	0.779042602	0.428756297
0.783735991	0.728694141	0.528492510
0.890027642	0.778761923	0.428083390
0.946323991	0.723072767	0.527569890
0.055585593	0.944408953	0.426382154
0.111519292	0.888471365	0.527308345
0.221988648	0.944175005	0.426925093
0.277585536	0.888494074	0.526553273
0.388855636	0.944177687	0.426926702
0.443627626	0.888477206	0.527314365
0.555490196	0.944411933	0.426389158
0.610993028	0.890644252	0.527478874
0.722292244	0.944781423	0.427158117
0.779032290	0.891404450	0.527543843
0.889154077	0.944777071	0.427154422
0.946315706	0.890639961	0.527476728
0.555331528	0.444335997	0.595058501

Supplementary Data 2 Structural file for finial state of Zn^{2+} diffusion on Zn (002) crystal plane without PAH/PAA multilayers.

POSCAR
1.0

15.9893999100	0.0000000000	0.0000000000
-7.9946999550	13.8472265133	0.0000000000
0.0000000000	0.0000000000	25.0000000000

Zn
73
Direct

0.055879809	0.111465424	0.427201241
0.112893730	0.057301074	0.527452230
0.222223714	0.111108057	0.426497459
0.278156191	0.055175900	0.527324617
0.388686329	0.110849760	0.427007824
0.444288105	0.055232994	0.526664019
0.555497587	0.110853106	0.427009135
0.610360205	0.055180844	0.527328610
0.722219229	0.111111790	0.426503062
0.777744830	0.057306442	0.527452707
0.888920128	0.111467972	0.427202463
0.945634425	0.057938792	0.527692735
0.055556595	0.277778000	0.426612288
0.110905290	0.222424835	0.527175307
0.221866250	0.277452767	0.427198440
0.276026905	0.220433682	0.527450621
0.387909532	0.276675135	0.428071439
0.443593115	0.220346972	0.527748883
0.554728150	0.276118934	0.428022027
0.610093892	0.220354840	0.527760684
0.722097754	0.276678294	0.428077251
0.777741909	0.220451608	0.527456582
0.888918281	0.277455151	0.427204043
0.944846034	0.222427323	0.527173817
0.055877961	0.444415450	0.427204430
0.110904172	0.388485759	0.527172685
0.221864522	0.444414377	0.427200556
0.275392592	0.387697369	0.527691662
0.387638241	0.443820000	0.428725362
0.438121557	0.383056611	0.528639555
0.554362416	0.442293704	0.428850830
0.610007226	0.386688024	0.529224038
0.721271336	0.442306876	0.428872824
0.778264403	0.383090913	0.528644919

0.889509857	0.443822473	0.428736389
0.945629716	0.387703240	0.527691483
0.056652583	0.611233652	0.428077608
0.112880811	0.555591524	0.527454436
0.222220570	0.611114442	0.426501662
0.276024133	0.555588901	0.527452767
0.387905687	0.611235738	0.428075641
0.438128978	0.555061936	0.528636217
0.554361582	0.612069786	0.428858757
0.595087171	0.547526598	0.522008598
0.722216666	0.611111224	0.433753937
0.785751104	0.547588587	0.522060990
0.891024590	0.612059116	0.428872228
0.950243652	0.555068374	0.528645277
0.057212178	0.778604209	0.428025424
0.112978250	0.723238587	0.527761638
0.222477764	0.777835190	0.427007824
0.278153121	0.722974420	0.527329922
0.388680220	0.777837694	0.427009940
0.443592370	0.723239839	0.527752519
0.554722309	0.778611958	0.428022563
0.610013783	0.723315537	0.529220819
0.721262574	0.778968811	0.428862661
0.785799146	0.738227963	0.522017717
0.891036868	0.778969169	0.428854167
0.946646094	0.723325312	0.529227853
0.056655589	0.945421517	0.428075016
0.112984285	0.889736712	0.527751029
0.222480655	0.944644928	0.427008390
0.278104246	0.889047027	0.526665628
0.388887048	0.944445014	0.427493095
0.444284528	0.889050007	0.526665092
0.555496097	0.944652438	0.427010953
0.610093713	0.889739811	0.527752280
0.722096086	0.945425749	0.428076714
0.778269768	0.895196736	0.528637350
0.889511645	0.945692658	0.428728521
0.950271428	0.895206809	0.528643429
0.722120583	0.611167490	0.595160127

Supplementary Data 3 Structural file for initial state of Zn^{2+} diffusion on Zn (002) crystal plane with PAH/PAA multilayers.

POSCAR
1.0

15.9893999100	0.0000000000	0.0000000000
-7.9946999550	13.8472265133	0.0000000000
0.0000000000	0.0000000000	30.0000000000

Zn	C	O	H	N
73	6	2	11	1

Direct

0.055760000	0.110579997	0.348639995
0.111409999	0.054790001	0.439529985
0.219990000	0.108649999	0.352250010
0.277300000	0.054170001	0.441810012
0.388429999	0.108730003	0.353579998
0.443650007	0.053690001	0.439359993
0.554660022	0.109420002	0.347570002
0.611519992	0.055280000	0.439420015
0.721979976	0.111139998	0.348679990
0.777869999	0.055080000	0.441769987
0.890049994	0.111309998	0.350849986
0.945630014	0.055760000	0.442499995
0.055470001	0.277509987	0.352200001
0.110349998	0.220730007	0.444260001
0.219290003	0.274589986	0.357400000
0.274650007	0.218349993	0.447659999
0.385789990	0.272909999	0.355049998
0.443949997	0.219070002	0.445089996
0.554979980	0.273570001	0.355300009
0.610509992	0.218970001	0.444299996
0.721759975	0.275620013	0.355589986
0.777729988	0.221479997	0.439619988
0.889360011	0.277790010	0.348379999
0.945339978	0.221680000	0.437750012
0.055670001	0.444909990	0.349359989
0.110529996	0.388500005	0.444319993
0.219650000	0.444110006	0.359479994
0.274040014	0.385600001	0.449750006
0.384469986	0.441790015	0.354600012
0.437070012	0.381339997	0.435099989
0.554329991	0.440400004	0.351060003
0.612389982	0.383399993	0.441960007
0.724460006	0.444359988	0.359699994
0.780219972	0.388240010	0.448320001

0.892279983	0.445179999	0.354420006
0.945469975	0.388859987	0.440490007
0.056650002	0.611559987	0.346890002
0.111070000	0.555410028	0.440750003
0.219679996	0.611140013	0.353789985
0.273849994	0.555299997	0.450450003
0.385850012	0.612720013	0.355560005
0.433530003	0.552730024	0.435990006
0.554009974	0.612479985	0.352250010
0.620329976	0.555700004	0.428869992
0.728299975	0.614000022	0.350160003
0.783770025	0.556599975	0.446130008
0.892480016	0.611859977	0.356059998
0.947369993	0.555899978	0.442770004
0.056919999	0.778110027	0.347979993
0.113020003	0.723169982	0.438540012
0.221880004	0.777920008	0.347059995
0.277429998	0.722199976	0.441000015
0.387860000	0.779389977	0.354279995
0.444040000	0.723360002	0.447050005
0.553950012	0.781459987	0.355540007
0.613319993	0.729820013	0.436479986
0.726530015	0.780650020	0.357279986
0.783860028	0.725639999	0.442990005
0.892530024	0.780340016	0.353879988
0.947820008	0.723429978	0.441080004
0.057009999	0.944949985	0.346769989
0.113150001	0.889330029	0.438389987
0.222069994	0.943929970	0.348019987
0.278409988	0.888890028	0.439790010
0.388339996	0.944280028	0.347039998
0.444299996	0.889060020	0.439339995
0.555159986	0.946259975	0.349500000
0.610570014	0.891359985	0.447160006
0.721719980	0.946330011	0.358530015
0.780979991	0.890900016	0.448890001
0.892929971	0.947499990	0.354620010
0.947009981	0.890550017	0.441489995
0.845605791	0.830326080	0.512696326
0.465590000	0.603139997	0.737240016
0.454930007	0.514670014	0.738640010
0.455949992	0.460070014	0.698790014
0.504909992	0.504499972	0.538969994
0.594039977	0.573440015	0.527019978

0.479189992	0.402559996	0.550090015
0.457300007	0.501630008	0.659619987
0.456389993	0.383320004	0.701960027
0.474460006	0.640120029	0.705550015
0.466120005	0.640829980	0.767639995
0.446920007	0.477039993	0.770089984
0.448240012	0.522930026	0.543980002
0.653029978	0.558019996	0.523150027
0.612219989	0.648389995	0.522180021
0.525430024	0.382030010	0.530950010
0.404110014	0.352030009	0.540180027
0.449209988	0.325239986	0.609990001
0.462689996	0.460070014	0.634819984
0.557579994	0.420690000	0.607749999
0.487210006	0.394820005	0.598800004

Supplementary Data 4 Structural file for finial state of Zn^{2+} diffusion on Zn (002) crystal plane with PAH/PAA multilayers.

POSCAR

1.0

15.9893999100	0.0000000000	0.0000000000
-7.9946999550	13.8472265133	0.0000000000
0.0000000000	0.0000000000	30.0000000000

Zn	C	O	H	N
73	6	2	11	1

Direct

0.055760000	0.110579997	0.348639995
0.111409999	0.054790001	0.439529985
0.219990000	0.108649999	0.352250010
0.277300000	0.054170001	0.441810012
0.388429999	0.108730003	0.353579998
0.443650007	0.053690001	0.439359993
0.554660022	0.109420002	0.347570002
0.611519992	0.055280000	0.439420015
0.721979976	0.111139998	0.348679990
0.777869999	0.055080000	0.441769987
0.890049994	0.111309998	0.350849986
0.945630014	0.055760000	0.442499995
0.055470001	0.277509987	0.352200001
0.110349998	0.220730007	0.444260001
0.219290003	0.274589986	0.357400000
0.274650007	0.218349993	0.447659999
0.385789990	0.272909999	0.355049998

0.443949997	0.219070002	0.445089996
0.554979980	0.273570001	0.355300009
0.610509992	0.218970001	0.444299996
0.721759975	0.275620013	0.355589986
0.777729988	0.221479997	0.439619988
0.889360011	0.277790010	0.348379999
0.945339978	0.221680000	0.437750012
0.055670001	0.444909990	0.349359989
0.110529996	0.388500005	0.444319993
0.219650000	0.444110006	0.359479994
0.274040014	0.385600001	0.449750006
0.384469986	0.441790015	0.354600012
0.437070012	0.381339997	0.435099989
0.554329991	0.440400004	0.351060003
0.612389982	0.383399993	0.441960007
0.724460006	0.444359988	0.359699994
0.780219972	0.388240010	0.448320001
0.892279983	0.445179999	0.354420006
0.945469975	0.388859987	0.440490007
0.056650002	0.611559987	0.346890002
0.111070000	0.555410028	0.440750003
0.219679996	0.611140013	0.353789985
0.273849994	0.555299997	0.450450003
0.385850012	0.612720013	0.355560005
0.433530003	0.552730024	0.435990006
0.554009974	0.612479985	0.352250010
0.620329976	0.555700004	0.428869992
0.728299975	0.614000022	0.350160003
0.783770025	0.556599975	0.446130008
0.892480016	0.611859977	0.356059998
0.947369993	0.555899978	0.442770004
0.056919999	0.778110027	0.347979993
0.113020003	0.723169982	0.438540012
0.221880004	0.777920008	0.347059995
0.277429998	0.722199976	0.441000015
0.387860000	0.779389977	0.354279995
0.444040000	0.723360002	0.447050005
0.553950012	0.781459987	0.355540007
0.613319993	0.729820013	0.436479986
0.726530015	0.780650020	0.357279986
0.783860028	0.725639999	0.442990005
0.892530024	0.780340016	0.353879988
0.947820008	0.723429978	0.441080004
0.057009999	0.944949985	0.346769989

0.113150001	0.889330029	0.438389987
0.222069994	0.943929970	0.348019987
0.278409988	0.888890028	0.439790010
0.388339996	0.944280028	0.347039998
0.444299996	0.889060020	0.439339995
0.555159986	0.946259975	0.349500000
0.610570014	0.891359985	0.447160006
0.721719980	0.946330011	0.358530015
0.780979991	0.890900016	0.448890001
0.892929971	0.947499990	0.354620010
0.947009981	0.890550017	0.441489995
0.658835173	0.825924695	0.512036145
0.465590000	0.603139997	0.737240016
0.454930007	0.514670014	0.738640010
0.455949992	0.460070014	0.698790014
0.504909992	0.504499972	0.538969994
0.594039977	0.573440015	0.527019978
0.479189992	0.402559996	0.550090015
0.457300007	0.501630008	0.659619987
0.456389993	0.383320004	0.701960027
0.474460006	0.640120029	0.705550015
0.466120005	0.640829980	0.767639995
0.446920007	0.477039993	0.770089984
0.448240012	0.522930026	0.543980002
0.653029978	0.558019996	0.523150027
0.612219989	0.648389995	0.522180021
0.525430024	0.382030010	0.530950010
0.404110014	0.352030009	0.540180027
0.449209988	0.325239986	0.609990001
0.462689996	0.460070014	0.634819984
0.557579994	0.420690000	0.607749999
0.487210006	0.394820005	0.598800004
